# Cluster of differentiation 8 and programmed cell death ligand 1 expression in triple-negative breast cancer combined with autosomal dominant polycystic kidney disease and tuberous sclerosis complex: a case report

**DOI:** 10.1186/s13256-019-2274-6

**Published:** 2019-12-24

**Authors:** Kenji Gonda, Takanori Akama, Takayuki Nakamura, Eiko Hashimoto, Naomi Kyoya, Yuichi Rokkaku, Yuko Maejima, Shoichiro Horita, Kazunoshin Tachibana, Noriko Abe, Tohru Ohtake, Kenju Shimomura, Koji Kono, Shigehira Saji, Seiichi Takenoshita, Eiji Higashihara

**Affiliations:** 1 Department of Genetics, Fukushima Medical University, 1 Hikarigaoka, Fukushima, Fukushima 960-1295 Japan; 2 Clinical Oncology Center, Fukushima Medical University Hospital, 1 Hikarigaoka, Fukushima, Fukushima 960-1295 Japan; 3 Department of Gastrointestinal Tract Surgery, Fukushima Medical University, 1 Hikarigaoka, Fukushima, Fukushima 960-1295 Japan; 4 Department of Bioregulation and Pharmacological Medicine, Fukushima Medical University, 1 Hikarigaoka, Fukushima, Fukushima 960-1295 Japan; 5 Daido Obesity and Metabolism Research Centre, 123 Daido, Naha, Okinawa, 902-0066 Japan; 6 Deptartment of Urology, Japan Community Healthcare Organization Nihonmatsu Hospital, 1-553 Naritamachi, Nihonmatsu, Fukushima, 964-8501 Japan; 7 Deptartment of Surgery, Japan Community Healthcare Organization Nihonmatsu Hospital, 1-553 Naritamachi, Nihonmatsu, Fukushima, 964-8501 Japan; 8 Department of Breast Surgery, Fukushima Medical University, 1 Hikarigaoka, Fukushima, Fukushima 960-1295 Japan; 9 President of Fukushima Medical University, Fukushima Medical University, 1 Hikarigaoka, Fukushima, Fukushima 960-1295 Japan; 10 Department of Polycystic Kidney Research, Kyorin University shool of Medicine, 6-20-2 Shinkawa, Mitaka, Tokyo, 181-8611 Japan

**Keywords:** ADPKD, *TSC2*/*PKD1* CGS, CD8^+^ T, PD-L1, CREBBP

## Abstract

**Background:**

Autosomal dominant polycystic kidney disease is defined as an inherited disorder characterized by renal cyst formation due to mutations in the *PKD1* or *PKD2* gene, whereas tuberous sclerosis complex is an autosomal dominant neurocutaneous syndrome caused by mutation or deletion of the *TSC2* gene. A *TSC2/PKD1* contiguous gene syndrome, which is caused by a chromosomal mutation that disrupts both the *TSC2* and *PKD1* genes, has been identified in patients with tuberous sclerosis complex and severe early-onset autosomal dominant polycystic kidney disease. The tumor tissue of patients with breast cancer with contiguous gene syndrome has a high mutation burden and produces several neoantigens. A diffuse positive immunohistochemistry staining for cluster of differentiation 8^+^ in the T cells of breast cancer tissue is consistent with neoantigen production due to high mutation burden.

**Case presentation:**

A 61-year-old Japanese woman who had been undergoing dialysis for 23 years because of end-stage renal failure secondary to autosomal dominant polycystic kidney disease was diagnosed as having triple-negative breast cancer and underwent mastectomy in 2015. She had a history of epilepsy and skin hamartoma. Her grandmother, mother, two aunts, four cousins, and one brother were also on dialysis for autosomal dominant polycystic kidney disease. Her brother had epilepsy and a brain nodule. Another brother had a syndrome of kidney failure, intellectual disability, and diabetes mellitus, which seemed to be caused by mutation in the *CREBBP* gene. Immunohistochemistry of our patient’s breast tissue showed cluster of differentiation 8 and programmed cell death ligand 1 positivity.

**Conclusions:**

Programmed cell death ligand 1 checkpoint therapy may be effective for recurrence of triple-negative breast cancer in a patient with autosomal dominant polycystic kidney disease and tuberous sclerosis complex.

## Background

Autosomal dominant polycystic kidney disease (ADPKD), which is defined as an inherited disorder characterized by renal cyst formation, is caused by dysfunction of polycystin 1 or polycystin 2, which leads to mutations in the *PKD1* or *PKD2* gene, respectively. Tuberous sclerosis complex (TSC) is an autosomal dominant neurocutaneous syndrome that is caused by mutation or deletion of the *Tuberous Sclerosis Complex 2* (*TSC2*) gene, which encodes for tuberin. The *PKD1* gene is adjacent to the *TSC2* gene on chromosome 16p13.3. *TSC2/PKD1* contiguous gene syndrome (CGS), which is caused by a chromosomal mutation that disrupts both the *TSC2* and *PKD1* genes, has been identified in patients with TSC and severe early-onset ADPKD [1]. Several reports characterized *TSC2/PKD1* CGS as a severe polycystic kidney growth with onset and end-stage renal failure at an early age [2–4]. Therefore, patients with constitutional deletions involving the *TSC2* and *PKD1* genes were suggested to have poor prognosis of their renal function. Here, we presented the case of a 61-year-old Japanease woman with ADPKD, TSC, and triple-negative breast cancer (TNBC). Gene deletion in tumor cells can lead to a high mutation burden, which can result in neoantigen production, as well as programmed cell death ligand 1 (PD-L1) expression. In the present case, immunohistochemical analysis indicated diffuse expressions of PD-L1 in the tumor and cluster of differentiation 8 (CD8)^+^ T around the tumor. Administration of an immune checkpoint inhibitor without chemotherapy may be considered when a patient who is undergoing dialysis develops cancer recurrence.

## Case presentation

**Fig. 1:**
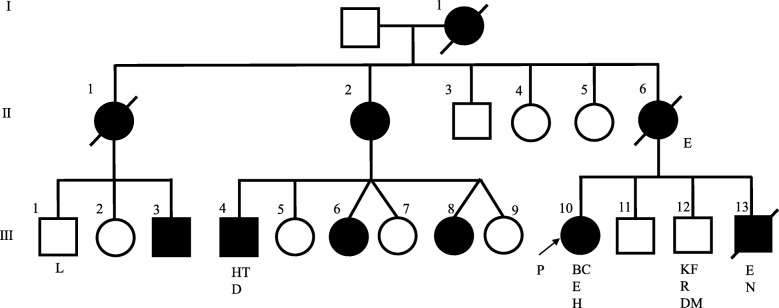
Reconstructed pedigree of the family with autosomal dominant polycystic kidney disease. *Squares* denote male family members, *circles* denote female family members, and *solid symbols* denote individuals affected by autosomal dominant polycystic kidney disease and undergoing kidney dialysis. The *arrow* denotes the proband, a *symbol with a slash* indicates a deceased person, and the diseases are listed below the symbols. *BC* breast cancer, *D* diverticulitis, *DM* diabetes mellitus, *E* epilepsy, *H* hamartoma, *HT* hypertension, *KF* kidney failure, *L* leukemia, *N* brain nodule, *P* proband, *R* intellectual disability

**Fig. 2:**
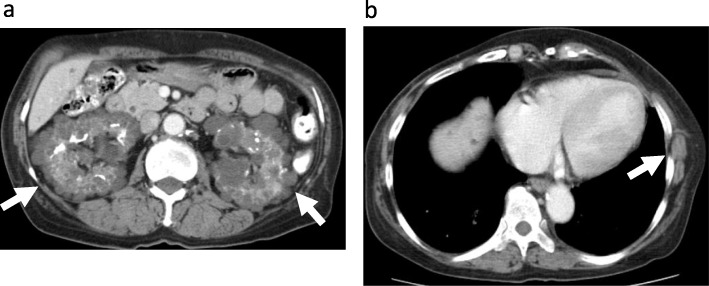
Radiologic findings in the proband. **a** Unenhanced computed tomography scan shows multiple kidney cysts (*arrow*) and **b** skin hamartoma (*arrow*)

A 61-year-old Japanese woman (proband, III-10) (Fig. 1) had been undergoing dialysis for 23 years for end-stage renal failure secondary to polycystic kidney disease (PKD) (Fig. 2a), which was diagnosed in 2003. She had childhood epilepsy, as well as hypertension and skin hamartoma (Fig. 2b). She temporarily changed her residence after the nuclear power plant leak that was caused by the 2011 Great East Japan Earthquake and Tsunami but later returned home. In 2015, she noticed stiffness in her right breast, which was biopsied and diagnosed as cancer, for which mastectomy with axillary lymph node dissection was performed. The pathologic diagnosis at that time was invasive ductal carcinoma, stage IIA: tumor (T) 2, node (N) 0, metastasis (M) 0, lymphatic invasion (Ly) 0, venous invasion (V) 0, estrogen receptor (ER) (−), progesterone receptor (PgR) (−), human epidermal growth factor receptor 2 (HER2) (−).

**Fig. 3:**
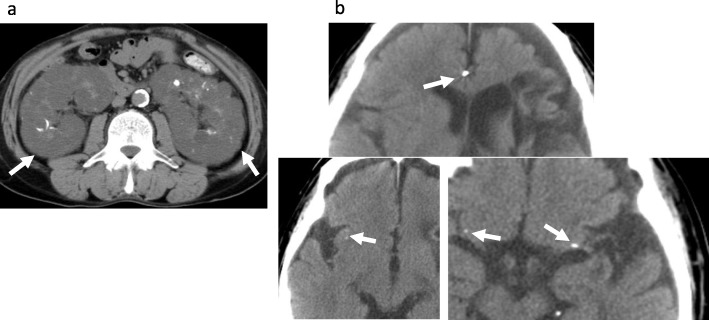
Radiologic findings in the proband’s brother. **a** Unenhanced computed tomography scan shows multiple kidney cysts (*arrow*) and **b** brain nodule (*arrow*)

Her family history (Fig. 1) revealed a brother (III-13) who was on dialysis for 19 years for ADPKD (Fig. 3a), epilepsy, and a brain nodule (Fig. 3b); he died at 54 years of age. She had a younger brother (III-12) who had kidney failure, intellectual disability, and diabetes mellitus (DM). Her mother (II-6) received dialysis for ADPKD, had epilepsy, and died at an unknown age. Our patient’s aunt (II-1) received dialysis for 16 years for ADPKD and died at 78 years of age; our patient’s aunt’s son (III-3) had ADPKD and was receiving dialysis. Another aunt (II-2) was on dialysis for ADPKD; her son (III-4) was receiving dialysis for ADPKD and had hypertension and diverticulitis; her twin daughters (III-6 and III-8, respectively) were receiving dialysis for ADPKD. Our patient’s grandmother (I-1) received dialysis for ADPKD and died at an unknown age. At present, our patient is on dialysis without any sign of recurrence, and we recommended her children be tested.

**Fig. 4:**
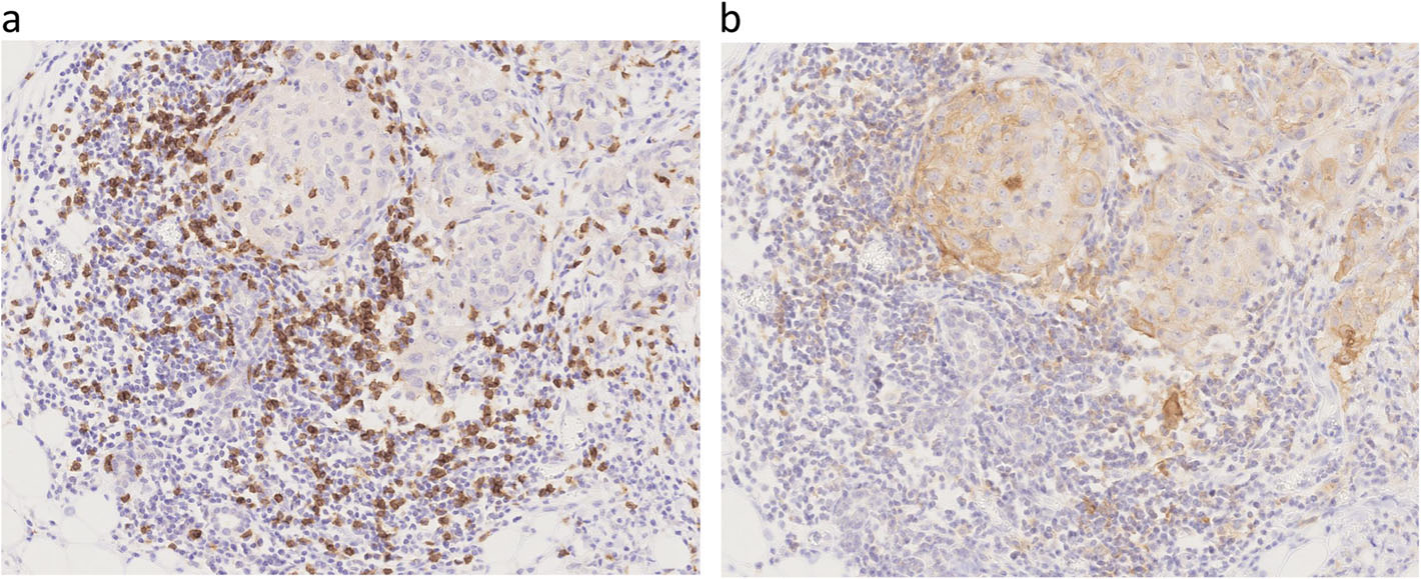
Immunohistochemistry for cluster of differentiation 8 and programmed cell death ligand 1. The infiltrating immune cells are immunohistochemically positive for **a** cluster of differentiation 8 and **b** programmed cell death ligand 1. **a** Cluster of differentiation 8 expression is detected using a mouse monoclonal antibody (clone C8/144B, catalog no. GA62361–2; Dako, Agilent Technologies, Santa Clara, CA, USA) (10 ×). **b** Programmed cell death ligand 1 expression is detected using a rabbit monoclonal antibody (clone E1L3N, catalog no. #13684; Cell Signaling Technology, Danvers, MA, USA) (10 ×)

Evaluation of the resected breast cancer tissue by immunohistochemistry showed CD8^+^ T cells on the tumor–stromal interface (Fig. 4a) and PD-L1 expression on the membrane of tumor cells (Fig. 4b). The increased CD8 expression seemed to be associated with the high PD-L1 expression.

## Discussion

### Mutation burden

The presence of *TSC2* and *PKD1* gene deletions should be considered in the clinical assessment of TSC in children with early-onset PKD and in all patients with ADPKD who develop end-stage renal failure prior to the fourth or fifth decade of life [5]. Dysfunction of the C-terminal tail of *PKD1* in *TSC2/PKD1* CGS was suggested to play a crucial role in renal prognosis [6].

Kleczko *et al.* suggested that T cells, specifically CD8^+^ T cells, had a functional role in ADPKD progression [7]. Therefore, the use of immune-oncology agents that target this pathway may represent a novel therapeutic approach for ADPKD [7]. First, patients with *TSC2/PKD1* CGS have high mutation burden and produce several neoantigens, which are produced by cancer cells that have not been previously recognized by the immune system. Second, the neoantigens have very high immunologic potential, because they evade thymic selection and central tolerance. Finally, multiple gene deletions, as well as monogenic deficiency, induce the production of cytotoxic immune cells, such as CD8^+^ T cells, and infiltration of CD8^+^ T cells around the tumor leads to neoantigen production. Endometrial cancer with polymerase epsilon ultra-mutation and microsatellite instability hypermutation has been shown to have an active tumor microenvironment (TME) with increased numbers of neoantigens and tumor-infiltrating lymphocytes (TILs) [8]. Interestingly, this patient exhibited an increased number of peripheral blood cells, such as myeloid-derived suppressor cells (MDSCs), which inhibit immunologic function. In our previous report, patients with stage II breast cancer had high blood levels of MDSC cells [9]. We speculated that CD8^+^ T cells may compete with MDSCs in the TME.

Increase in the number of both CD8^+^ T cells and TILs is associated with survival. Moreover, the high PD-L1 expression in patients with relatively high CD8^+^ T cell count indicates an adaptive immune mechanism. In the present case, the diffuse positive immunohistochemistry staining for CD8 and PD-L1 in breast cancer tissue was consistent with neoantigen production due to high mutation burden. In addition, in a murine model of pulmonary lymphangioleiomyomatosis, increased PD-L1 expression was observed in Tsc2-null lesions [10].

### TNBC and immunotherapy

In a previous report on TNBC [11], T cells were shown to decrease in density as they moved in from the boundary of tumor cell clusters; this density increased as the T cells approached the center. Although tumor mutational burden was evaluated in that study, it was not prognostic and did not correlate with PD-L1 or CD8 gene expression. In patients with TNBC, PD-L1 is expressed mainly on tumor-infiltrating immune cells, rather than on the tumor cells, and can inhibit the anticancer immune response. Therefore, inhibition of programmed cell death 1 (PD-1) and PD-L1 may be a useful treatment strategy. Unresectable locally advanced or metastatic TNBC is an aggressive disease with poor outcomes. Nanoparticle albumin-bound (nab)-paclitaxel may enhance the anticancer activity of atezolizumab. Based on the results of available trials [12, 13], this combination of chemotherapy and immunotherapy clearly benefited patients with PD-L1-positive tumors and may be considered for recurrent cases. On the other hand, first-line pembrolizumab monotherapy was shown to have a manageable safety profile and durable antitumor activity therapy for patients with PD-L1-positive metastatic TNBC [14]. Merck announced the phase 3 KEYNOTE-119 trial on Keytruda® (pembrolizumab) as monotherapy for the second-line or third-line treatment of patients with metastatic TNBC [15]. However, that trial did not meet its pre-specified primary endpoint of superior overall survival (OS), compared with that of chemotherapy with capecitabine, eribulin, gemcitabine, or vinorelbine. As per the study protocol, the other endpoints were not formally tested because the primary endpoint of OS was not met.

At present, research to identify biomarkers of good response to immunotherapy is still ongoing. Some of the possible biomarkers that are being explored include TILs, tumor PD-L1 expression, and tumor mutational burden. However, all the mechanisms involved in immunotherapy have not been completely understood, thereby making it difficult to identify a biomarker that can broadly work for all the approved immunotherapies. For patients with PD-L1-positive tumors (that is, ≥ 1% PD-L1 expression on tumor-infiltrating immune cells), first-line treatment with nab-paclitaxel and atezolizumab was recommended, when available [16]. Further characterization of the immune microenvironment may highlight the targets for immune-based therapy of TNBC.

To develop an immunohistochemical scoring algorithm that includes PD-L1 expression for both tumor and immune cells (that is, combined positive score) [17], the varying clinicopathologic features and survival outcomes of TNBC need to be determined according to the different histologic subtypes. Medullary carcinoma and apocrine adenocarcinoma have excellent prognosis, whereas mixed lobular–ductal carcinoma and metaplastic carcinoma are the most aggressive subtypes [18]. TNBC can be categorized into six different molecular subtypes that are characterized by distinct biological features. The different molecular clusters described were basal-like 1, basal-like 2, immunomodulatory, mesenchymal, mesenchymal stem-like, and luminal androgen receptor. These TNBC molecular subtypes can be targeted by specific therapies [19] and have substantial genomic heterogeneity with distinct patterns of genomic alterations and putative underlying driver mutations [20].

In fact, identification of biomarkers that can guide treatment decisions in TNBC remains a clinically unmet need. Understanding the mechanisms that drive resistance is the key to the development of novel therapeutic strategies that can help prevent metastatic disease and, ultimately, improve survival in this patient population [21].

### *TSC2* and breast cancer

Mutation in the *TSC2* gene alters the function of the TSC1/TSC2 complex, so that it no longer functions as a guanosine-5'-triphosphate (GTP) ase-activating protein. As a result, the mammalian target of rapamycin (mTOR) activity is promoted, which increases protein synthesis. This increased protein synthesis in TSC was thought to be associated with the clinical manifestations, such as dysplasia, and tumorigenesis due to abnormal cell proliferation and neovascularization.

At a glance, breast cancer appears to be unrelated to PKD. The *endonuclease III-like 1* (*NTHL1*) gene may be involved in DNA repair and is present in the 68-bp region, downstream of the *TSC2* gene. The *TSC2* gene is located upstream of the *NTHL1* gene by 5′-to-5′ (head-to-head) placement, and the promoters of both *NTHL1* and *TSC2* genes overlap. The *NTHL1* gene was said to be causative of multiple cancers, including breast cancer [22]. Furthermore, the gene is located in a 3′-to-3′ (tail-to-tail) orientation with the *solute carrier family 9 isoform A3 regulatory factor 2* (*SLC9A3R2*) gene [23]. The *SLC9A3R2* gene of the Na+/H+ exchange carrier controlling element is present in the 8778-bp region upstream of the *TSC2* gene. These genes were thought to be located on chromosome 16p13.3, from the centromere side, in the order of *TSC2*, *NTHL1*, and *SLC9A3R2* [24]. The *SLC9A3R2* gene is broadly expressed in fat tissue and is a housekeeping gene that consistently showed low variance as the normalizing genes in the microarray profiles of all breast cancer datasets [25].

### Polycystic kidney disease type 1 (PKD1) and Rubinstein–Taybi (RTS) syndrome

One of our patient’s younger brothers had ADPKD and died at a young age, whereas the other younger brother had intellectual impairment, which was most likely RTS [26]. RTS comprises multiple congenital anomalies and is characterized by intellectual disability, postnatal growth deficiency, microcephaly, broad thumbs and halluces, and dysmorphic facial features. It is caused by heterozygous mutation in the gene that encodes the transcriptional coactivator cyclic adenosine monophosphate (cAMP)-response element-binding protein (CREB) binding protein (CREBBP) on chromosome 16p13.3 [27]. Inhibition of hypothalamic CREBBP results in profound obesity, impaired glucose homeostasis, and increased food intake [28]. Surprisingly, one of our patient’s younger brothers has DM, is stout, and may have *CREBBP* gene deficiency. The unique feed–forward signaling loop of cAMP−CREB−glycogen synthase kinase 3 beta (GSK3β)−cAMP is potentially self-sustaining, and blocking it at any point would break this vicious cycle and significantly reduce PKD progression [29]. Therefore, the younger brother was unlikely to have PKD.

## Conclusions

In this patient with TNBC complicated with ADPKD, there were high mutation burden, increased CD8 and PD-L1 expressions in the breast cancer tissue, and neoantigen production. If recurrence occurs, PD-L1 immune checkpoint inhibitors may be effective.

## Data Availability

The datasets used and/or analyzed during the current study are available from the corresponding author on request.

## Abbreviations

ADPKD: Autosomal dominant polycystic kidney disease
cAMP: Cyclic adenosine monophosphate
CD8: Cluster of differentiation 8
CGS: Contiguous gene syndrome
CREB: Cyclic adenosine monophosphate-response element-binding protein
CREBBP: Cyclic adenosine monophosphate-response element-binding protein binding protein
DM: Diabetes mellitus
MDSC: Myeloid-derived suppressor cell
nab: Nanoparticle albumin-bound
*NTHL1*: *Endonuclease III-like 1*
OS: Overall survival
PD-1: Programmed cell death 1
PD-L1: Programmed cell death ligand 1
PKD: Polycystic kidney disease
*PKD1*: *Polycystic kidney disease 1*
*PKD2*: *Polycystic kidney disease 2*
RTS: Rubinstein–Taybi syndrome
*SLC9A3R2*: *Solute carrier family 9 isoform A3 regulatory factor 2*
TIL: Tumor-infiltrating lymphocyte
TME: Tumor microenvironment
TNBC: Triple-negative breast cancer
TSC: Tuberous sclerosis complex
*TSC2*: *Tuberous Sclerosis Complex 2*
Tsc2: Tuberous sclerosis complex 2
